# Diabetes mellitus does not alter mortality or hospitalisation risk in patients with newly diagnosed heart failure with preserved ejection fraction: Time to rethink pathophysiological models of disease progression

**DOI:** 10.1177/14791641231224241

**Published:** 2024-04-16

**Authors:** John Gierula, Sam Straw, Charlotte A Cole, Judith E Lowry, Maria F Paton, Melanie McGinlay, Klaus K Witte, Peter J Grant, Stephen B Wheatcroft, Michael Drozd, Thomas A Slater, Richard M Cubbon, Mark T Kearney

**Affiliations:** 1 Leeds Institute of Cardiovascular and Metabolic Medicine, 573267 University of Leeds, Leeds, UK; 2 Medical Clinic 1, University Hospital Aachen, Aachen, Germany

**Keywords:** Heart failure, preserved, ejection fraction, mortality, diabetes

## Abstract

**Introduction:**

Type 2 diabetes is a common and adverse prognostic co-morbidity for patients with heart failure with reduced ejection fraction (HFrEF). The effect of diabetes on long-term outcomes for heart failure with preserved ejection fraction (HFpEF) is less established.

**Methods:**

Prospective cohort study of patients referred to a regional HF clinic with newly diagnosed with HFrEF and HFpEF according to the 2016 European Society of Cardiology guidelines. The association between diabetes, all-cause mortality and hospitalisation was quantified using Kaplan-Meier or Cox regression analysis.

**Results:**

Between 1st May 2012 and 1st May 2013, of 960 unselected consecutive patients referred with suspected HF, 464 and 314 patients met the criteria for HFpEF and HFrEF respectively. Within HFpEF and HFrEF groups, patients with diabetes were more frequently male and in both groups patients with diabetes were more likely to be treated with β-adrenoceptor antagonists and angiotensin converting enzyme inhibitors. After adjustment for age, sex, medical therapy and co-morbidities, diabetes was associated with increased mortality in individuals with HFrEF (HR 1.46 95% CI: 1.05–2.02; *p* = .023), but not in those with HFpEF (HR 1.26 95% CI 0.92–1.72; *p* = .146).

**Conclusion:**

In unselected patients with newly diagnosed HF, diabetes is not an adverse prognostic marker in patients with HFpEF, but is in HFrEF.

## Introduction

Over 23 million people worldwide are estimated to have chronic heart failure (CHF)^1,2^ with around half of these having normal, or near normal, left ventricular ejection fraction (LVEF).^3,4^ This disorder is now defined as heart failure with preserved left ventricular ejection fraction (HFpEF). Major advances in our understanding of the natural history^5^ and pathophysiology^6^ of heart failure with reduced left ventricular ejection fraction (HFrEF), have led to the development and widespread use of disease modifying pharmacological^7–9^ and device-based therapies.^10,11^ As a result, survival of patients with HFrEF has substantially improved over the last three decades.^12,13^ However, the same cannot be said of patients with HFpEF.

It is well established that type 2 diabetes mellitus is an adverse prognostic feature in patients with HFrEF and that this higher risk persists despite optimal medical therapy.^14,15^ A number of studies have suggested that diabetes is also a risk factor for all-cause mortality in patients with HFpEF,^16–20^ however these studies employed inconsistent definitions of HFpEF, excluded patients with common comorbidities^21^ and of particular importance, elderly patients with HFpEF were under represented.^16^ Diabetes has also previously been shown to increase risk of heart failure hospitalisation in HFpEF.^22^ In the present report we examine the effect of diabetes on long-term mortality and hospitalisation in consecutive unselected patients with newly diagnosed HFpEF or HFrEF, as defined by the European Society of Cardiology (ESC) guidelines, seen by a regional heart failure service.^23^

## Methods

As described in our earlier publication,^24^ we conducted a prospective cohort study in which we collected data on consecutively referred patients with suspected heart failure between 1st May 2012 and 1st May 2013. Patients with signs or symptoms of CHF, as well as elevated natriuretic peptides (NT-proBNP ≥125 pg/mL) were assessed in a specialist heart failure clinic, and classified as having HFpEF, HFrEF or not as having CHF according to the 2016 ESC recommendations. This manuscript presents a *post* hoc analysis of the original study. Upon arrival at the clinic, demographic details, height and weight were recorded. We collected data on cardiovascular and non-cardiovascular co-morbidities known to adversely affect the prognosis of heart failure, as well as medical therapy for heart failure and relevant co-morbidities. Patients underwent clinical assessment by specialist nurses. The presence of diabetes was determined by either a diagnosis from primary care records, or the receipt of oral hypoglycaemic medication or insulin. Blood pressure was taken (right arm recumbent), electrocardiography and trans-thoracic echocardiography performed, and patients were reviewed by one of two consultants with a specialist interest in heart failure (KKW, MTK). Venous blood was collected for assessment of renal function, and hematological parameters. Samples were analysed in the local hospital chemical pathology laboratories. Estimated glomerular filtration rate (eGFR) was calculated using the Modification of Diet in Renal Disease method.^25^

### Natriuretic peptide measurement

NT-pro-BNP concentration was measured in samples taken in primary care using the Immulite 2000 assay (Siemens Healthcare Diagnostics, Camberley, UK) in the biochemistry laboratory at Leeds Teaching Hospitals NHS Trust. The interbatch coefficient of variation was 8.9% at 350 pg/mL and 5.9% at 4100 pg/mL.

### Echocardiography

Two-dimensional trans-thoracic echocardiography was performed by echocardiographers (JG, JEL, MP) blinded to NT-pro-BNP measurements and patient characteristics. Left ventricular (LV) dimensions, LV ejection fraction (LVEF), LV mass, left atrial (LA) and LV Doppler measurements were calculated according to the American Society of Echocardiography (ASE) and European Association of Cardiovascular Imaging (EACI) guidelines,^26^ LV mass and LA volume were indexed to body surface area (BSA). We also measured Cardiac Contractility Index as a surrogate of cardiac contractility using the formula systolic blood pressure/left ventricular end systolic volume index (SBP/LVESVi).^27^

### Patient classification

Patients with HFpEF were required to have signs and symptoms of HF, elevated levels of natriuretic peptides - NT-proBNP ≥125 pg/mL, LVEF ≥50%, relevant structural heart disease (left atrial volume index (LAVI) > 34 mL/m^2^ and/or a left ventricular mass index (LVMI) ≥115 g/m^2^ for males and ≥95 g/m^2^ for females) and/or abnormal diastolic filling. Patients with HFrEF had signs and symptoms of HF, NT-proBNP ≥125 pg/mL and LVEF <50%. We did not subdivide those with LVEF<50% further.

### Follow up and mortality

The primary outcome for this study was all-cause mortality. Vital status data were collected using linked Hospital Episode Statistics and Office of National Statistics mortality data following S251 ethical approval (CAG 8-03(PR1)/2013). All surviving patients had a minimum follow-up of 6 years, with final censorship date 30th April 2019. We also evaluated mode-specific death as a secondary outcome, classified as previously described^12,28^ as either: (1) HF death; (2) Other cardiovascular death and (3) Other non-cardiovascular causes of death; as well as all-cause and mode specific hospitalisation.

Hospitalisation data were collected from clinical event databases detailing all hospital admissions. All non-elective admissions experienced before death or study censorship were included, and characterised by two investigators according to their time from study recruitment, duration, and primary cause within four major categories: (1) HF hospitalisation; (2) Other cardiovascular hospitalisation (e.g. arrhythmia or acute coronary syndrome, without decompensated HF); (3) Infection-related hospitalisation; (4) Other non-cardiovascular hospitalisation (non-cardiovascular cause excluding infection-related). HF hospitalisation was defined as new onset or worsening signs and symptoms of heart failure with evidence of fluid overload requiring at least 24 h hospitalisation and the use of intravenous diuretics, as we have previously published.^12,24^ Infection-related hospitalisation was defined as infection being the primary reason for hospitalisation with documented source (or suspected source), accompanied by deteriorating symptoms, signs (e.g. pyrexia, tachycardia, hypotension, tachypnoea, confusion) and laboratory indices (e.g. elevated inflammatory markers, with microbiological, serological, and/or imaging evidence) resulting in treatment with antimicrobial therapy, as previously described.^29^

### Statistical analysis

All statistical analyses were performed using IBM SPSS statistics version 27 (IBM Corporation, Armonk, NY, USA). Normal distribution of data was confirmed using skewness and kurtosis tests. Continuous data are presented as mean ± standard deviation (SD), or median [interquartile range] and categorical data are shown as number (percentage). Groups were compared using two-sided Student t-tests or ANOVA for parametric continuous data, Kruskal-Wallis tests for non-parametric continuous data and two-sided Pearson χ^2^ tests for categorical data. Unadjusted survival was displayed by Kaplan-Meier plot with differences between groups determined by log-rank tests. We used Cox proportional hazards regression to determine the association between diabetes status and all-cause mortality in HFrEF and HFpEF, with adjusted regression models including age, sex, and equivalent doses of bisoprolol and ramipril. In all analyses, statistical significance was defined as *p* < .05.

## Results

Between 1st May 2012 and 1st May 2013, 982 patients with suspected heart failure and NT-proBNP ≥125 pg/mL were assessed. Of these, 22 had insufficient quality echocardiographic images to assess cardiac structure and function. Following echocardiography and clinical assessment, 476 (49%) were classified as having HFpEF, 311 (32%) as having HFrEF, and 182 (19%) as not having CHF. Our final dataset therefore consisted of 778 patients, of whom 434 (55.8%) were female and had a mean age of 83.0 ± 9.2 years.

### Patient characteristics

Patients with HFpEF were older than those with HFrEF (83.6 ± 8.6 v 81.9 ± 10.0 years; *p* < .001), more often female (65.1% v 41.8%; *p* < .001), more likely to have a history of hypertension (75.6% v 56.6%; *p* < .001), and less likely to have a history of ischaemic heart disease (22.5% v 37.0%; *p* < .001). The majority of patients referred were over the age of 80 with 337 (72%) and 200 (64%) in the HFpEF and HFrEF cohorts respectively. Median follow up was 5.6 (2.8-6.4) years and in total the cohort provided 3549 patient years of follow-up. Median [IQR] HbA1c was 44.0 [40.0 – 52.5] mmol/mol v 47.0 [42.0 – 56.0] mmol/mol; *p* < .001 for HFpEF and HFrEF respectively, with the difference in HbA1c between patients with and without diabetes similar in both groups (Table 1). Patients with diabetes were younger (80.3 ± 9.7 v 64.1 ± 8.8 years; *p* = .041), more often male (57.2% v 39.3%; *p* < .001), had higher body mass index (26.3 ± 6.0 v 23.0 ± 4.8 kg/m^2^), were more likely to have ischaemic heart disease (38.1% v 24.5%; *p* < .001), hypertension (76.3% v 64.8%; *p* = .002), and had more impaired renal function (eGFR 62.3 ± 21.2 v 66.3 ± 18.7 mL/min/1.73 m^2^; *p* = .005).

**Table 1.** Characteristics of patients presenting to secondary care based on the European Society of Cardiology Guidelines for the diagnosis of heart failure with preserved ejection fraction (HFpEF) and heart failure with reduced ejection fraction (HFrEF), with and without diabetes.

	All	Heart failure preserved ejection fraction *n* = 467		*p* Value	Heart failure reduced ejection fraction *n* = 311		*p* Value
		No diabetes	Diabetes		No diabetes	Diabetes	
Number per group	778	355 (76%)	112 (24%)		208 (67%)	103 (33%)	
Demographics and previous medical history							
Age (years)	83.0 ± 9.2	84.4 ± 8.3	81.6 ± 9.3	0.003	83.5 ± 9.6	78.9 ± 10.0	<0.001
Male sex	344 (44%)	108 (30%)	55 (49%)	<0.001	113 (54%)	68 (66%)	0.049
White-European ethnicity	735 (95%)	340 (95.8%)	97 (86.6%)	0.006	207 (99%)	91 (88%)	<0.001
Body mass index (kg/m^2^)	23.9 ± 5.4	28.5 ± 7.2	32.3 ± 6.1	<0.001	27.4 ± 5.2	30.9 ± 7.5	<0.001
Ischaemic heart disease	220 (28%)	69 (19%)	36 (32%)	0.005	69 (33%)	46 (45%)	0.048
Hypertension	529 (68%)	255 (72%)	98 (88%)	0.001	110 (53%)	66 (64%)	0.061
Atrial fibrillation	279 (36%)	132 (37%)	40 (36%)	0.779	66 (32%)	41 (40%)	0.158
Chronic obstructive pulmonary disease	114 (14.7%)	53 (15%)	11 (10%)	0.17	31 (15%)	19 (18%)	0.42
Echocardiographic and haemodynamic data							
Systolic blood pressure (mmHg)	140.1 ± 23.5	144.0 ± 21.9	142.5 ± 23.0	0.553	135.2 ± 25.7	134.1 ± 21.8	0.716
Heart rate (beats per minute)	75.7 ± 16.7	73.9 ± 14.7	73.5 ± 14.0	0.829	78.8 ± 19.3	77.4 ± 19.3	0.548
Left ventricular ejection fraction (%)	48.4 ± 11.9	56.2 ± 4.0	56.0 ± 4.1	0.651	36.7 ± 10.4	36.7 ± 9.3	0.990
Cardiac contractility index (SBP/LVESVi)	4.6 ± 1.9	5.5 ± 1.7	5.2 ± 1.4	0.098	3.2 ± 1.3	3.2 ± 1.4	0.931
Laboratory data							
HbA1c (mmol/mol)	45.0 [41.0 – 55.0]	41.0 [38.0 – 43.0]	54.0 [48.0 – 63.0]	<0.001^a^	42.0 [39.0 – 44.0]	56.0 [50.0 – 66.0]	<0.001^a^
Estimated glomerular filtration rate (mL/min/1.73 m^2^)	65.2 ± 19.5	66.7 ± 18.9	61.7 ± 20.6	0.017	65.6 ± 18.4	62.9 ± 22.0	0.250
Haemoglobin (g/L)	128.7 ± 19.4	129.6 ± 19.7	123.1 ± 16.6	0.002	130.4 ± 19.7	128.0 ± 19.4	0.306
NT-proBNP (pg/mL), median [IQR]	1054 [510–2562]	838 [440 – 1745]	854 [416 – 1578]	0.885^a^	1939 [736 – 4196]	1275 [563 – 3559]	0.075^a^
Medical therapy							
Beta blocker prescription	432 (56%)	185 (52%)	73 (65%)	0.015	110 (53%)	65 (63%)	0.087
Bisoprolol equivalent dose (mg)	2.8 ± 3.5	2.7 ± 3.4	3.7 ± 3.8	0.009	2.4 ± 3.1	3.2 ± 3.7	0.029
ACE/ARB prescription	471 (61%)	184 (52%)	90 (80%)	<0.001	125 (60%)	72 (70%)	0.002
Ramipril equivalent dose (mg)	3.3 ± 3.8	2.9 ± 3.6	4.7 ± 4.2	<0.001	2.9 ± 3.5	4.2 ± 3.9	0.002
Diuretic prescription	370 (48%)	142 (40%)	57 (51%)	0.042	114 (55%)	57 (55%)	0.929
Furosemide equivalent dose (mg)	0.0 [0.0 – 40.0]	0.0 [0.0 – 40.0]	20.0 [0.0 – 40.0]	0.074^a^	20.0 [0.0 – 40.0]	20.0 [0.0 – 40.0]	0.358^a^
Insulin prescription	35 (4%)	0	18 (16%)	n/a	0	16 (16%)	n/a
Metformin prescription	103 (13%)	0	54 (48%)	n/a	0	49 (48%)	n/a
Sulphonyl prescription	54 (7%)	0	28 (25%)	n/a	0	25 (24%)	n/a
Glitazone prescription	9 (1%)	0	6 (5%)	n/a	0	3 (3%)	n/a

^a^Mann-Whitney U test.

Data are n (%), mean ± SD or median [IQR].

SBP/LVESVi: systolic blood pressure/left ventricular end systolic volume index; ACE: Angiotensin-converting enzyme inhibitor; ARB: Angiotensin II receptor blocker.

### Heart failure with preserved ejection fraction

Descriptive data for this group are presented in Table 1. Type 2 diabetes was present in 24% (*n* = 112) of patients, which was treated with the following strategies: sulfonylureas 25%, metformin 54%, insulin 18%, and thiazolidinediones 5%. HFpEF patients with diabetes were more likely to receive β-adrenoceptor blockers and angiotensin converting enzyme inhibitors (ACEI) than HFpEF patients without diabetes, and at higher doses.

### Heart failure with reduced ejection fraction

Descriptive data for this group are presented in Table 1. Type 2 diabetes was present in 33% (*n* = 103) of patients, which was treated with the following strategies: sulfonylureas 24%, metformin 49%, insulin 16%, thiazolidinediones 3%. HFrEF patients with diabetes were more likely to receive higher doses of β-adrenoceptor blockers and ACEI as HFrEF patients without diabetes.

### Mortality

**Figure 1.**
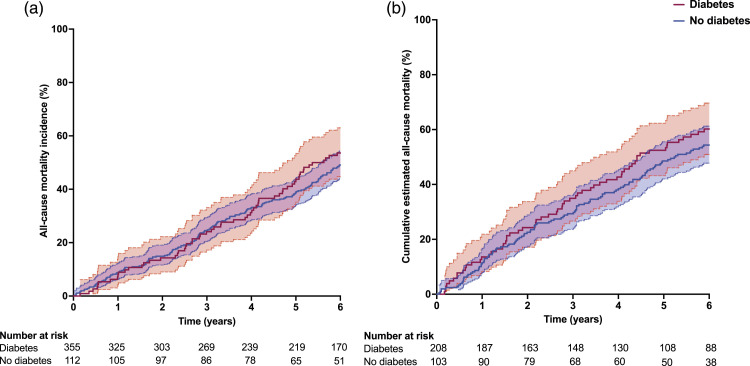
Kaplan Meier survival curves (unadjusted) comparing all-cause mortality incidence for patients with or without diabetes for those with: (a) HFpEF and (b) HFrEF.

**Figure 2.**
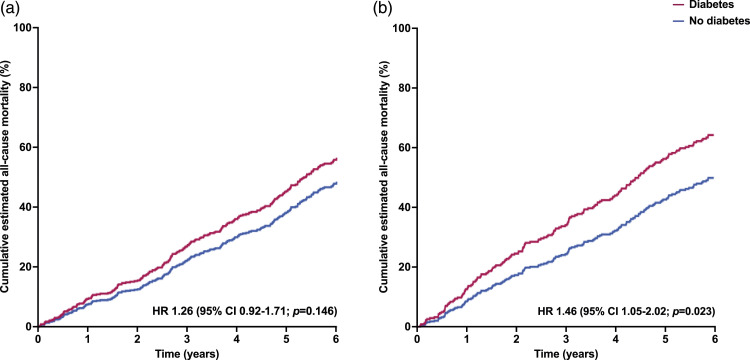
Estimated cumulative all-cause mortality comparing patients with or without diabetes adjusted for age, sex and treatment for those with: (a) HFpEF and (b) HFrEF.

During the follow-up period a total of 424 (54%) patients died. The cumulative incidence of all-cause mortality was 92.1%, 76.0%, and 61.0%, for patients with HFpEF, and 89.1%, 69.5% and 50.8%, for patients with HFrEF at 1, 3, and 5 years, respectively (Figure 1). In unadjusted Cox regression analysis diabetes was not associated with adverse prognosis in patients with HFrEF (HR 1.13 [95% CI 0.84–1.54]; *p* = .42) or HFpEF (HR 1.09 [95% CI 0.81–1.45]; *p* = .57). However, after also accounting for prognostically relevant baseline characteristics (ischaemic heart disease, hypertension, and renal function) and medical therapy (equivalent dose of bisoprolol and ramipril) diabetes was associated with an increased risk of all-cause mortality in patients with HFrEF (HR 1.46 [95% CI: 1.05 – 2.02]; *p* = .023), but not in HFpEF (HR 1.26 [95% CI: 0.92 – 1.72]; *p* = .146) (Table 2 and Figure 2). Diabetes was not associated with increased risk of death in any of our mode-specific death analyses in patients with HFpEF or HFrEF.

**Table 2.** Absolute and adjusted hazard of diabetes and mortality in patients with heart failure and preserved ejection fraction (HFpEF) and heart failure with reduced ejection fraction (HFrEF).

Variable	HFpEF			HFrEF		
	HR	95% CI	*p* value	HR	95% CI	*p* value
Diabetes (unadjusted)	1.088	0.814 – 1.454	0.570	1.134	0.836 – 1.539	0.419
Diabetes (adjusted for age and sex)	1.197	0.893 – 1.605	0.228	1.336	0.974 – 1.833	0.073
Diabetes (adjusted for age, sex, Bisoprolol equivalent dose and Ramipril equivalent dose)	1.252	0.928 – 1.691	0.142	1.486	1.079 – 2.048	0.015
Diabetes (adjusted for age, sex, Bisoprolol equivalent dose, Ramipril equivalent dose and ischaemic heart disease)	1.280	0.945 – 1.733	0.111	1.487	1.079 – 2.049	0.015
Diabetes (adjusted for age, sex, Bisoprolol equivalent dose, Ramipril equivalent dose and, hypertension)	1.270	0.940 – 1.716	0.119	1.482	1.074 – 2.044	0.017
Diabetes (adjusted for age, sex, Bisoprolol equivalent dose, Ramipril equivalent dose and estimated glomerular filtration rate)	1.210	0.893 – 1.639	0.218	1.461	1.056 – 2.022	0.022
Diabetes (adjusted for age, sex, Bisoprolol equivalent dose, Ramipril equivalent dose, ischaemic heart disease, hypertension and estimated glomerular filtration rate)	1.261	0.922 – 1.724	0.146	1.459	1.053 – 2.021	0.023

### Hospitalisation

At the end of the follow-up period a total of 500 (64%) patients had been hospitalised at least once, with 301 (65%) all-cause hospitalisations in patients with HFpEF and 199 (64%) in those with HFrEF. A diagnosis of diabetes did not result in a higher rate of hospitalisation for any cause in HFpEF (77 (69%) v 224 (63%); *p* = .276) or HFrEF (68 (66%) v 131 (63%); *p* = .599) and did not increase the burden of hospitalisation (Table 3). Cause-specific analyses showed that a significantly higher number of patients with diabetes and HFrEF were admitted for infection related hospitalisation (38 (37%) v 54 (26%); *p* = .047) than patients with HFpEF (38 (34%) v 108 (30%); *p* = .485) (Table 3). The hazard ratio for hospitalisation was greater in patients with diabetes and HFrEF – age/sex adjusted HR 1.77 (95% CI: 1.15 – 2.72); *p* = .009 than people with HFpEF and diabetes – age/sex adjusted HR 1.34 (95% CI: 0.92 – 1.96); *p* = .127.

**Table 3.** Burden of all-cause hospitalisation, and number of people admitted for mode-specific hospitalisation, in patients with heart failure with preserved ejection fraction (HFpEF) or heart failure with reduced ejection fraction (HFrEF), with and without diabetes.

	Heart failure preserved ejection fraction *n* = 467		*p* Value	Heart failure reduced ejection fraction *n* = 311		*p* Value
	No diabetes	Diabetes		No diabetes	Diabetes	
	355 (76%)	112 (24%)		208 (67%)	103 (33%)	
Total number of hospitalisations per patient	1.0 [0.0 – 3.0]	1.0 [0.0 – 5.0]	0.111^a^	1.0 [0.0 – 3.0]	1.0 [0.0 – 3.0]	0.739^a^
Total days spent in hospital during follow-up period	20.0 [8.0 – 42.75]	26.0 [9.5 – 39.5]	0.435^a^	23.0 [8.0 – 47.0]	20.0 [6.0 – 39.0]	0.356^a^
Average duration of hospitalisation	7.8 [4.0 – 14.0]	7.6 [4.6 – 13.0]	0.864^a^	7.0 [4.0 – 15.5]	4.0 [7.2 – 10.0]	0.394^a^
Any hospitalisation	224 (63%)	77 (69%)	0.276	131 (63%)	68 (66%)	0.636
Heart failure hospitalisation	34 (10%)	14 (13%)	0.375	24 (12%)	16 (16%)	0.322
Other cardiovascular hospitalisation	55 (15%)	24 (21%)	0.144	37 (18%)	20 (19%)	0.727
Any infection-related hospitalisation	108 (30%)	38 (34%)	0.485	54 (26%)	38 (37%)	0.047
Other non-cardiovascular hospitalisation	132 (37%)	41 (37%)	0.912	66 (32%)	41 (40%)	0.158

Data are median [IQR] or n (%); ACE: Angiotensin-converting enzyme inhibitor; ARB: Angiotensin II receptor blocker.

^a^Kruskal Wallis test.

## Discussion

The present report provides important new information for health care professionals and patients with heart failure and diabetes. Our most important findings are that in our patients with a new diagnosis of heart failure diabetes was not an adverse prognostic marker in patients with carefully diagnosed HFpEF, whereas diabetes is associated with an almost 50% adjusted increase in the risk of death of people with HFrEF, confirming both our previous work^14^ and that of others.^15^ Furthermore, we also confirm our previous work demonstrating that diabetes increases the risk of infection-related hospitalisation in HFrEF,^29^ whereas the present data suggest that this is not the case for HFpEF. By including all confirmed cases referred to the single referral centre for suspected heart failure serving a population of approximately 750,000 people, with a mean age reflecting a more “real world” heart failure population than is usual in a clinical trial,^30^ our data offer a truly representative assessment of how diabetes interacts with heart failure at a population level.

### Insights into potential mechanisms underlying the differential prognostic impact of diabetes in patients with HFrEF and HFpEF

The current study was not designed to examine disease mechanisms. However, both groups had good glycaemic control according to current guidelines,^31^ therefore the differential association of diabetes with mortality in patients with HFrEF versus HFpEF suggests diabetes may adversely interact with specific elements of the HFrEF phenotype. We recently^32^ quantified the association between escalating doses of the heart failure disease modifying agents, β-adrenoceptor antagonists and ACEI, in patients with diabetes and HFrEF. We showed a significant benefit of incremental doses of ACEI in patients with HFrEF and diabetes and intriguingly demonstrated a potentially greater benefit from β-adrenoceptor antagonists in patients with both diabetes and HFrEF than those with HFrEF alone suggesting greatest benefit in those at highest risk.

It is well established that heightened activation of neurohumoral systems is a key mechanism underpinning the pathophysiology of HFrEF. Diabetes, which is also a condition of sympathoactivation,^33^ is likely therefore to act in synergy with the effect of HFrEF on these pathways. Unlike HFrEF, treatment of HFpEF with neurohumoral antagonists has not led to an improvement in mortality,^34–37^ suggesting the presence of different disease mechanisms in HFpEF. However, we also observed that diabetes was associated with an increased risk of infection-related hospitalisation in HFrEF, and so some of the increased risk associated with diabetes in this population may be related to reasons aside from the adverse effect on cardiovascular physiology.

A second broad mechanism thought to underpin the progression of HFrEF is adverse left ventricular remodelling secondary to disruption of left ventricular size and shape.^6^ We examined left ventricular dimensions in patients with HFrEF and HFpEF with and without diabetes. Interestingly, and again contrary to current thinking, patients with diabetes and HFpEF and HFrEF had larger left ventricular volumes. In the setting of HFpEF this finding goes against current pathophysiological paradigms suggesting that the disadvantageous changes in left ventricular geometry, so important in HFrEF, do not impact unfavourably on prognosis in HFpEF patients with diabetes. Rather, other factors such as the presence of atrial fibrillation may lead to the development of progressive heart failure in this population, as we have previously reported.^24^

### Clinical Implications of the Present Study

In the present study over 50% of patients with HF per se were over 80 years of age, in line with previous reports in unselected patients newly referred with suspected CHF. Despite this, almost 50% of these patients were still alive 6 years later, with similar figures for patients with HFpEF or HFrEF. This was despite markers of risk such as elevated natriuretic peptides, which were numerically higher in those with HFrEF. This finding might imply more severe disease. However, contrasting between different classifications of heart failure may be problematic, as natriuretic peptides are released in response to wall stress which parallels left ventricular remodelling, not usually seen in HFpEF.

These data suggest that careful treatment of patients with HF can now afford even elderly patients a good life expectancy that in patients with HFrEF and diabetes may be improved with optimisation of disease modifying agents. It is also relevant that diabetes does not seem to be associated with a deleterious effect on mortality in older patients with HFpEF, which may have less influence on hospitalisation or survival as other co-morbidities such as hypertension in older patients. This suggests future clinical trials may more appropriately focus on younger patients or indeed those with HFrEF in whom almost all studies demonstrate an unfavourable effect of diabetes on outcomes.^38^

It is also notable that diabetes is associated with a more modestly increased risk of mortality in our study than in others focussed on younger cohorts; this phenomenon has previously been reported,^39,40^ and may represent a shorter duration of diabetes or a distinct phenotype of diabetes present in later life. Prior studies which have suggested diabetes is associated with an adverse prognosis in HFpEF were primarily conducted in patients who had previously been hospitalised and were therefore at an increased risk of poor outcomes, and usually not classified according to the ESC criteria.^18,41,42^

### Study Strengths and Limitations

Our investigation is the first to describe the interaction between HFpEF and diabetes on mortality and hospitalisation in patients with rigorously assessed diagnoses according to contemporary international guidelines. The ESC guidelines for the diagnosis of HFpEF^23^ mandate the presence of clinical, biochemical and strictly defined echocardiographic criteria to make the diagnosis of HFpEF. The present study was able to perform and evaluate the long-term implications of this diagnosis in the presence of diabetes.

Our study sample was referred with a suspected diagnosis of heart failure from primary care with symptoms, signs and elevated natriuretic peptides, unlike previous analyses of randomised clinical trials or historical cohorts where the diagnosis of HFpEF was often made on an arbitrary value of LVEF.^21^ The provision of guideline directed medical therapy was lower than what might be anticipated, however reflects background therapy at the point of referral to the heart failure service. Moreover, our study sample represents patients with a *de novo* diagnosis of heart failure, and unlike clinical trials, our sample did not exclude patients based on comorbidities such as renal impairment, anaemia or chronic airflow limitation - an important consideration especially when the pathophysiology of HFpEF is thought to be closely linked to multiple co-morbidity.^21^ A further strength of our study is the mean age of over 80 years; unlike retrospective analyses of clinical trials, this is truly representative of the heart failure population by including all suspected heart failure in a population of approximately 750,000.

However, a number of potential limitations of our study should also be considered. Our study, was conducted at a single centre, which may limit generalization to other regions, although the diverse characteristics of the area served by our centre, recently described by ourselves,^43^ mitigates against this potential limitation. A relatively small sample size is an additional limitation, and our finding that diabetes status was not associated with an adverse prognosis in HFpEF may be the result of a type 2 error. We did not account for competing risks when analysing hospitalisation risk, and our findings may underestimate the effect of diabetes on hospitalisation in HFrEF due to a competing risk of death. The observational design of our study does not allow us to provide a mechanism for the effect of diabetes on mortality in patients with HFrEF. We did not analyse the effect of diabetes on HF of different aetiologies although we have previously demonstrated a similar prognostic impact of diabetes on HFrEF patients with ischaemic or non-ischaemic aetiology.^14^ We did not collect data regarding revascularisation or device implantation during follow-up, and other co-morbidities known to be associated with prognosis not reported here. Finally, sensitivity analyses according to diabetes duration or severity were not possible due to a small sample size and it is feasible that those with HFpEF and less well managed diabetes would have a more adverse prognosis.

### Conclusion

This study is the first to use a prospectively recruited cohort of unselected patients with heart failure to examine the effect of diabetes on long term mortality in patients with clinical, biochemical and echocardiographic data to support the diagnosis of HFpEF. In our patients, we make the important observation that patients with diabetes and HFpEF do not share the elevated risk associated with HFrEF and diabetes. These findings will help plan resource allocation, clinical practice and future clinical trials.
